# Lambert-Eaton myasthenic syndrome in association with Sjögren’s syndrome: a case report

**DOI:** 10.1007/s13760-026-03099-5

**Published:** 2026-06-04

**Authors:** Pavel Potuznik, Jakub Vejskal, Kamila Smolikova, Marek Peterka

**Affiliations:** 1 Department of Neurology, Faculty of Medicine and University Hospital Pilsen, Charles University, Pilsen, Czech Republic; 2 Department of Neurology, Faculty of Medicine, University Hospital Hradec Kralove, Charles University in Prague, Prague, Czech Republic

## Background

Lambert-Eaton myasthenic syndrome (LEMS) is a rare paraneoplastic neuromuscular disorder characterized by proximal muscle weakness, hyporeflexia or areflexia, and autonomic symptoms. It is an autoimmune presynaptic neuromuscular junction disorder caused by the loss of functional P/Q-type voltage-gated calcium channels (VGCCs P/Q-type). The diagnosis of LEMS relies on a threefold approach: clinical features, electromyography (EMG), and anti-VGCC antibody serology. LEMS is a clinically significant early indicator of potential cancer. Up to 60% of cases occur as a paraneoplastic disorder, most commonly in association with small cell lung cancer (SCLC) [1]. The remaining cases are idiopathic and non-tumorous but are frequently linked to underlying autoimmune disease, including autoimmune thyroid disease, diabetes mellitus, rheumatoid arthritis, and Sjögren’s syndrome, in some cases [2–4].

## Case presentation

A 70-year-old nonsmoking woman with Sjögren’s syndrome, characterized by sicca syndrome, positive anti-Ro52 (SS-A) antibodies, anti-centromere protein B antibodies, and leukopenia, presented with progressive symptoms. Her difficulties began in May 2023, when she experienced trouble climbing stairs. Empirical treatment with oral corticosteroids initially resolved her symptoms, but upon discontinuation, the symptoms recurred in January 2024. Initially, she noticed impaired gait, which progressively worsened from April 2024, accompanied by general weakness with ongoing urinary infection. However, antibiotic treatment for the infection did not result in significant improvement. By July 2024, her condition had deteriorated further, requiring the use of a low walker for mobility, with worsening arm weakness and an inability to perform activity such as throwing.

Objectively, there was proximal muscle weakness (the Medical Research Council (MRC) summary score 52/60) with predominance in the lower limbs with concomitant areflexia/hyporeflexia of the deep tendon reflexes. A facilitation phenomenon of proprioceptive reflexes was observed after isometric contraction, with the most notable improvement seen in the C5/6 reflex after biceps brachii contraction. She exhibited hip girdle weakness, waddling gait, and difficulty rising from a chair independently.

An MRI of the thighs revealed no evidence of edema but showed generalized muscle hypotrophy. Muscle enzyme levels were within normal limits. Myositis-specific and myositis-associated autoantibodies, with the exception of anti-Ro52, were negative.

**Fig. 1:**
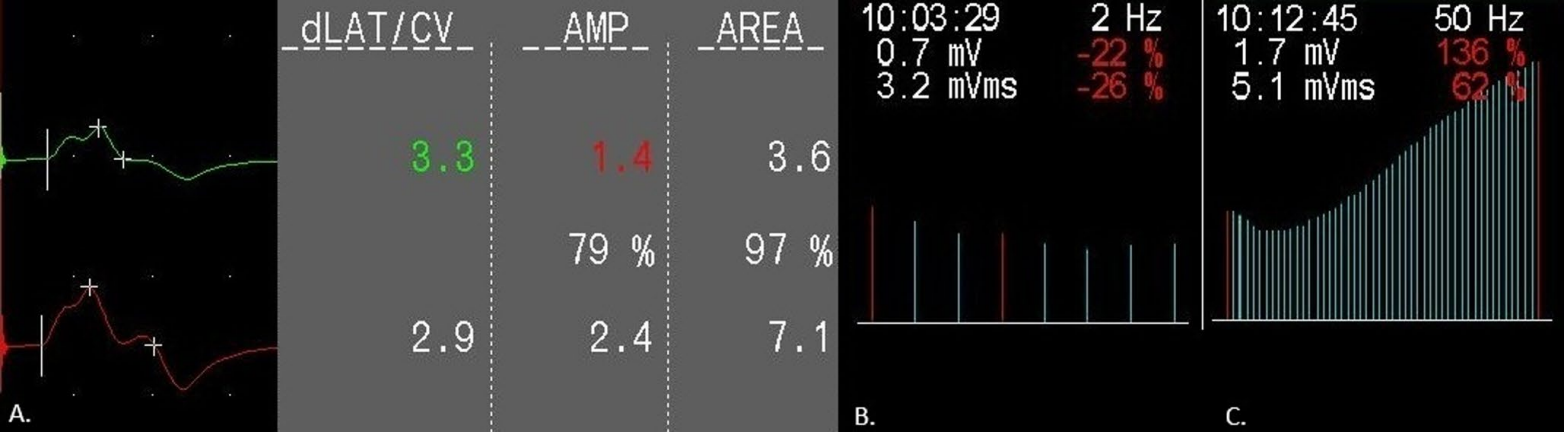
The electrophysiological study of the compound muscle action potential (CMAP) amplitude in the abductor pollicis brevis muscle: [**A**] before (superior CMAP) and after (inferior CMAP) a brief maximum voluntary contraction: increment of 79%; [**B**] decrement of 22% in the repetitive stimulation at 2 Hz; [**C**] increment of 136% after 50 repetitive stimulations at 50 Hz

Nerve conduction studies revealed low-amplitude compound muscle action potentials (CMAPs) with a post-exercise increment ≥ 60–100% (depending on criteria), highly suggestive of LEMS. Needle EMG demonstrated short-duration and small motor unit action potentials similar to myogenic findings without spontaneous activity. Repetitive nerve stimulation confirmed presynaptic neuromuscular transmission impairment, with an increment during high-frequency stimulation (Fig. 1). These findings supported the diagnosis of LEMS.

Cerebrospinal fluid analysis showed intrathecal synthesis, with 6 IgG oligoclonal bands (4 without serum correlate). Anti-VGCCs P/Q-type antibodies were detected in serum at a level of 124 pmol/L (normal < 30, radioimmunoassay), confirming the diagnosis of LEMS. Other antineuronal antibodies were negative. Additional serological findings included anti-centromere protein A and B antibodies and anti-Ro52 antibodies, consistent with the patient’s known Sjögren’s syndrome.

Treatment included a combination of pyridostigmine (amifampridine is not available in the Czech Republic), prednisone (initial dose 40 mg with taper to 5 mg) and cyclic administration of intravenous immunoglobulin (IVIg – 2 g/kg with reduction to 0.4 g/kg every 4–5 weeks), which led to significant clinical improvement (MRC 58/60). The patient regained the ability to walk independently, with only mildly myopathic gait.

Repeated PET/CT scans of the trunk (the last one in 2026) showed no evidence of malignancy, supporting the diagnosis of non-paraneoplastic LEMS associated with Sjögren’s syndrome.

### Discussion and conclusion

LEMS almost invariably begins as subacute proximal muscle weakness, especially in the lower limbs. Hyporeflexia or areflexia is another common symptom, which improves functionally after physical effort due to the facilitation phenomenon caused by Ca²⁺ accumulation at the nerve terminal, leading to increased acetylcholine release into the synaptic cleft [1]. Autonomic symptoms, such as dry mouth, are present in approximately 90% of LEMS cases [5]. In our patient, sicca syndrome was initially attributed solely to Sjögren’s syndrome, and the primary working diagnosis was myopathy (overlap myositis) without other autonomic symptoms. However, classic electrophysiological abnormalities, including low CMAP amplitude, a decrement in CMAP with low-rate stimulation, and an increment in CMAP following brief maximal voluntary contraction or high-frequency repetitive nerve stimulation, confirmed the diagnosis of LEMS. Furthermore in LEMS, myogenic findings without spontaneous activity may be observed on needle EMG.

LEMS is classified as a high-risk paraneoplastic phenotype. Anti-VGCC P/Q-type antibodies are detected in nearly 90% of patients, although their presence is not mandatory for diagnosis. These antibodies are found in both paraneoplastic and non-paraneoplastic forms of the disease. Conversely, anti-SOX1 are strongly associated with LEMS and SCLC [5]. In our case, paraneoplastic antibodies, including anti-SOX1, were negative, but antibodies associated with Sjögren’s syndrome (anti-centromere protein A and B, and anti-Ro52) were confirmed. Intrathecal synthesis is also unusual for classic LEMS but is compatible with concomitant autoimmune disease.

We searched PubMed and EMBASE up to May 1988 using the terms ‘Lambert-Eaton’, ‘Sjogren’, ‘Sjögren’s syndrome’ and ‘Sicca’ and found only two prior case reports. One involved paraneoplastic LEMS, while the second described non-paraneoplastic LEMS associated with discoid lupus erythematosus [3, 4]. Both cases were reported in the Japanese population. To our knowledge, our case is the first reported in a Caucasian individual.

Treatment of LEMS involves both symptomatic and immunomodulatory approaches. Symptomatic treatment primarily includes the potassium channel blocker 3,4-diaminopyridine (amifampridine), which is highly effective but is currently not available in the Czech Republic. Therefore, pyridostigmine has been used instead, though it is less effective. Immunomodulatory treatment options include prednisone with azathioprine, IVIg, or plasma exchange [1, 5].

In all cases of LEMS, it is essential to search for a paraneoplastic etiology. PET/CT imaging is indicated, and oncological treatment becomes the priority if a tumor is detected. If the initial tumor screening is negative, it should be repeated every 4–6 months for the next 2 years [5]. Although most LEMS cases have a paraneoplastic etiology, there are instances of non-paraneoplastic LEMS associated with other autoimmune diseases. Our case represents only the third reported association between LEMS and Sjögren’s syndrome. This raises the hypothesis of whether Sjögren’s syndrome in the context of LEMS might be underdiagnosed, as both conditions share overlapping autonomic symptoms. Further case reports and studies are needed to clarify this association.

## Data Availability

No datasets were generated or analysed during the current study. Available from corresponding author on reasonable request.
